# Behind Closed Membranes: The Secret Lives of Picornaviruses?

**DOI:** 10.1371/journal.ppat.1003262

**Published:** 2013-05-02

**Authors:** Alexsia L. Richards, William T. Jackson

**Affiliations:** Department of Microbiology and Molecular Genetics, Medical College of Wisconsin, Milwaukee, Wisconsin, United States of America; The Fox Chase Cancer Center, United States of America

If there is one truism that often opens a review or talk about picornaviruses, it is that the entire replication cycle of these simple positive-strand RNA viruses takes place in the cytosol. This statement is usually made to directly contrast picornaviruses with retroviruses or DNA viruses that require transport to the nucleus. However, the statement is meant quite literally. Some enveloped RNA viruses enter organelles to bud from the cellular secretion pathway, while other RNA viruses replicate their genomes in tightly controlled organelle invaginations. In contrast, every step in the replication of picornaviruses, once the genome has entered the cytosol, has long been thought to take place directly in the cytoplasm or on the cytoplasmic face of membranous structures [1].

Recently in *PLOS Pathogens*, we reported that inhibiting acidification of cellular vesicles reduces yield of the type picornavirus, poliovirus, by 90% or more [2]. These studies were an extension of our work on poliovirus subversion of the autophagic pathway, a degradative cell stress and homeostasis pathway that promotes the replication of several RNA viruses [3], [4]. The hallmark of autophagy is the presence of double-membraned vesicles with cytoplasmic contents, which acidify and fuse with lysosomes to facilitate degradation of their contents [5]. We suspect for several reasons that mature autophagosomes are the acidic vesicles crucial for normal levels of infectious virus production.

We were able to specifically show that acidic compartments of the cell promote the maturation cleavage of capsid protein VP0, a poorly understood event that is the final step in generation of an infectious virion [6], [7]. For these reasons, and data from the literature described below, we have proposed a model in which maturation of virions inside autophagosomes is promoted or accelerated by the acidic environment. There are alternate explanations, of course. Vesicles could be acting as ion sinks, for example, maintaining a more neutral balance in the cytoplasm. Inhibiting acidic vesicles might also be altering cellular organelles in a way that inhibits the virus. However, we think the most likely explanation is that virions find themselves in the interior of autophagosomes.

The role of autophagosomes in poliovirus replication has long been controversial. We and others long believed the cytoplasmic face of these vesicles to be a likely site of genomic RNA replication. This was primarily due to the localization of multiple virus-encoded RNA replication proteins to the autophagosome membrane [8], [9]. However, a competing hypothesis emerged, primarily from the labs of Kurt Bienz and Ellie Ehrenfeld. Their work showed that viral RNA replication proteins localized to single-membraned vesicles containing components of the cellular COPII machinery [10], [11]. Complicating these observations is the sensitivity of PV RNA replication to inhibition of the Arf family of small GTPases [11]. Arfs are key regulators of the cellular secretory pathway. In their activated form, Arfs regulate the recruitment of coat proteins COPI and clathrin to newly formed vesicles [12], [13]. Arfs are activated by the activity of guanine nucleotide exchange factors [12]. PV recruits both Arfs and their activating GEFs to the sites of RNA replication [14], [15]. Like poliovirus, coxsackievirus B3 (CVB3) recruits both Arf1 and its activating GEFs to the sites of RNA replication [16]. For coxsackievirus, it was shown that Phosphatidylinositol-4-kinase III beta (PI4KIIIbeta), a downstream effector of Arf1 signaling, also localizes to the sites of RNA replication, and the activity of this lipid kinase is essential for CVB3 replication [16]. However, while CVB3 and PV both subvert autophagy, the two viruses have very different effects on the autophagic pathway, so here we will focus on what is known for poliovirus [2], [16]–[18].

The first inkling that the COPII and autophagy hypotheses might not be mutually exclusive came from the Ehrenfeld lab, which performed a series of EM tomography experiments over a time course of PV infection [19]. Single-membraned vesicles predominate in the first few hours of infection. Later, convoluted invaginations of the single-membraned vesicles are observed. This results in structures morphologically similar to the crescent-shaped phagophore, which is the precursor to the double-membraned autophagosome [20]. By 6 hours post-infection, double-membraned vesicles predominate. Viral proteins and active RNA replication is associated with both types of structure. However, the exponential phase of RNA replication occurs when predominantly single-membraned vesicles are present. The authors proposed a model in which single-membraned vesicles morph into double-membraned vesicles, and suggested that the single-membraned vesicles are the primary sites of viral genome replication.

**Figure 1:**
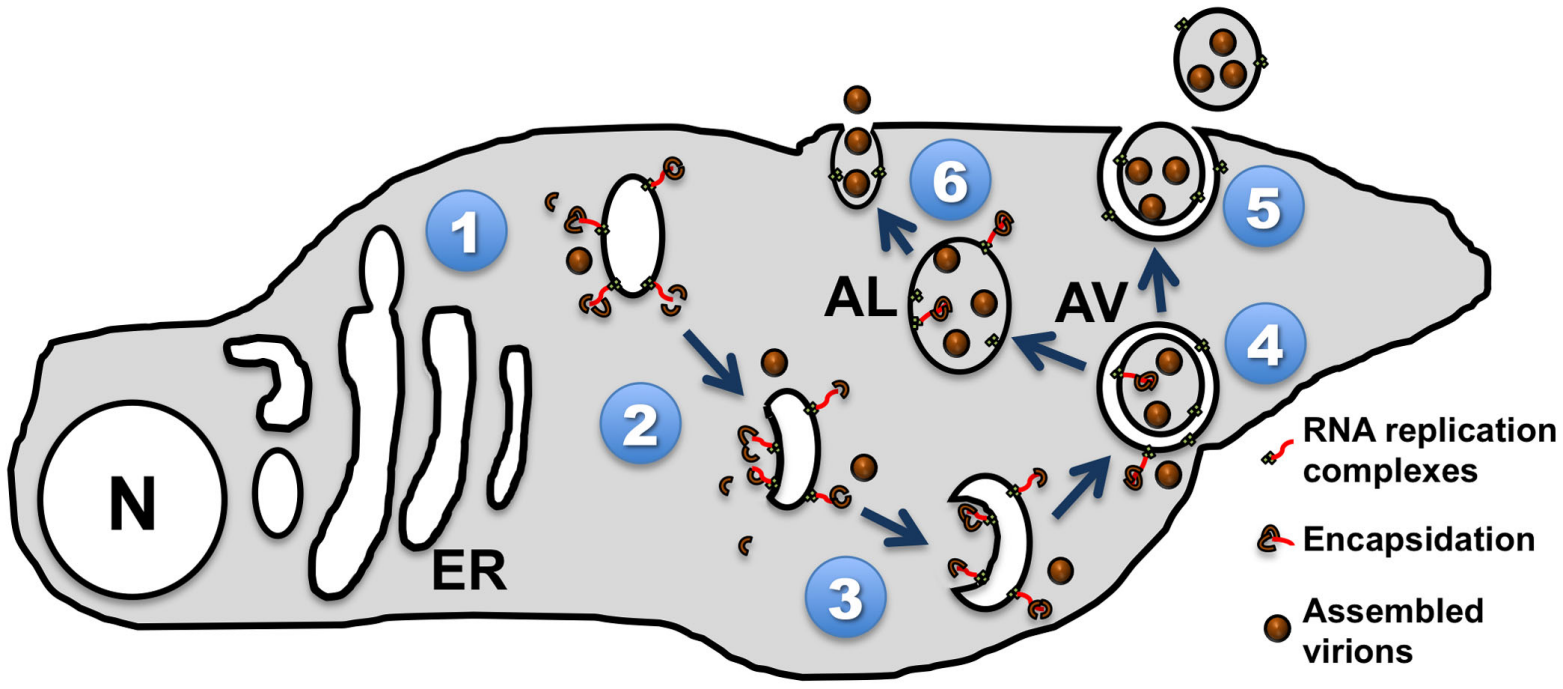
Model of picornavirus interactions with cellular membranes and vesicles. (**1**) Viral proteins induce rearrangement of cellular membranes, including COPII-like vesicles budding from the endoplasmic reticulum (ER). (**2**) The cytoplasmic faces of these vesicles act as a physical anchor for viral genomic RNA replication. (**3**) As single-membraned vesicles begin to invaginate into bowl-like structures, RNA replication and encapsidation are equally likely to occur inside or outside (**4**) the resultant double-membraned autophagic vesicles (AV). This is consistent with electron micrographs found in Horne and Nagington's study from 1959, and the Dales et al. study of 1965 [19], [24]. (**5**) These double-membraned vesicles could fuse with the plasma membrane to release a single-membraned virus-filled vesicle. (**6**) Our favored hypothesis, in which AVs mature into autolysosomes (AL) with single membranes. These vesicles would then fuse with the plasma membrane as suggested by electron micrographs in Dunnebacke et al. in 1969, thereby releasing naked virions into the extracellular space. N = nucleus.

In our recent *PLOS Pathogens* paper, we showed that inhibition of autophagosome formation, which potentially inhibits formation of the single-membraned precursor vesicles, reduces viral RNA replication [2]. Our data, along with that of the Ehrenfeld and Bienz labs, have led us to propose a unified model in which both vesicle populations play a pivotal role in infectious virus production (Figure 1). While the single-membraned precursor vesicle is essential for genome replication, the subsequent double-membraned vesicle promotes the later steps in virus production, specifically provirion maturation.

The cytoplasmic surface of a single-membraned vesicle is topologically uniform. Thus, all viral replication on that surface will be exposed to the cytoplasm. As the vesicle invaginates and fuses to form the double-membraned vesicle, a portion of what was once the outer membrane will now face the interior of the vesicle. This event explains how the synthesis of viral particles may take place in both environments. Accordingly, when PV-infected cells are examined by electron microscopy (EM) at 6 hours post-infection, viral particles are observed both free in the cytoplasm and trapped within double-membraned vesicles [21].

Therefore, we have substantial, albeit circumstantial, evidence that many potentially infectious poliovirions may find themselves inside double-membraned, autophagosome-like vesicles. The topology of these vesicles presents a unique challenge to a nonenveloped virus. Three lipid bilayers stand between the virus and the extracellular milieu. If these virions are destined to infect other cells, how do they exit both the vesicle and the cell?

Picornaviruses are the simplest human viruses, physically consisting of a positive-sense RNA genome and a capsid [22]. The current model for exit of picornaviruses from cells is disruption of the plasma membrane resulting in a lysis event that releases waiting cytoplasmic virions [23]. However, if a cell full of virus-containing double-membraned vesicles lyses, releasing the vesicles, then two lipid bilayers remain between the virions and the receptors on the surface of the next cell. Such double-membraned virus-containing structures have not been identified, although it is possible that autophagic vesicles released into the extracellular milieu would be unstable and short-lived, giving rise to naked, nonenveloped virions capable of engaging with the poliovirus receptor on a neighboring cell.

The autophagy field provides another possible answer. As autophagosomes mature into autolysosomes, they lose one membrane through an unknown mechanism [24], [25]. We have shown that autophagosomes formed during PV infection are capable of maturing into active autolysosomes [2]. It is likely that these autolysosomes contain virus components along with any other cytosolic contents. Poliovirus, a virus of the gut, would likely be capable of surviving the acidic, degradative autolysosome environment. This would leave only two lipid bilayers between the virus and the extracellular space: the remaining autophagic membrane, and the plasma membrane. If a single-membraned autolysosome were to fuse with the plasma membrane, then naked virions, which have been bathed in an acidic environment to promote their maturation into infectious virus, would be released from the cell.

Images from the classic literature support aspects of our model. In 1959, Horne and Nagington used electron microscopy to observe PV-infected, lysed HeLa fragments [26]. They found membrane-bound structures containing what appear to be virions in various stages of assembly, suggesting that virion formation can take place in a cellular compartment. In 1969, Dunnebacke et al. performed EM on PV-infected HeLa and chorion cells and found striking images of virus in membrane compartments, some of which appear to be in the act of fusing with the plasma membrane to release virus to the extracellular space [27]. These data led to a model, as described by Koch and Koch in 1985, in which “infectious virus is released through vacuoles which fuse with the plasma membrane. After several hours, virions escape the cell in a burst, when host cells lyse and die” [28].

This idea of vesicle-mediated release has been lost in the more recent literature, and the standard model for exit of poliovirus from the cell almost always describes a complete disruption of the plasma membrane, releasing all cytoplasmic contents. However, multiple experiments have shown that significant amounts of infectious virions can also be released in the absence of, or prior to, lysis, with the effect depending on factors such as the cell line used or the level of autophagic signaling during infection [29]–[31]. Inhibiting autophagosome formation, or movement of autophagic vesicles within the cell, dramatically reduces the prelytic release of infectious PV [31]. Another picornavirus, hepatitis A virus, rarely induces lysis of infected cells while still producing progeny virus [32]. These data led to the suggestion that autophagy could be related to a noncanonical secretion pathway for picornaviruses [8]. Since that suggestion, autophagy has been shown to function as a secretion pathway for the cellular protein Acb1 [33]–[35].

If we envision autophagic secretion as a fusion event between a double-membraned vesicle and the plasma membrane, this would result in the release of virions surrounded by a single membrane: in essence, an enveloped virus. This is not likely to be a common event, and based on what we know about poliovirus, a lipid envelope would not be productive, since it would prevent interactions between the virus capsid proteins and the cellular poliovirus receptor. However, we now have evidence that step in formation of a picornavirus may occur in a cellular compartment. We can imagine that whatever advantage is gained by maturing inside an autophagosome-like vesicle could be parlayed into an evolutionary link between nonenveloped and enveloped viruses.

First, we imagine a mutant virus that somehow promotes double-membraned autophagosome fusion with the plasma membrane instead of autophagosome maturation. Fusion of a double-membraned vesicle with the plasma membrane would result in release of “enveloped viruses.” This would result in difficulties, as the envelope—the former inner autophagic membrane—would not necessarily be equipped to facilitate a fusion event with the plasma membrane of a neighboring cell. However, there are clear advantages to being enveloped, including enhanced immune evasion, and if the enveloped virion managed to enter another cell, then the envelope-promoting mutant genome might propagate. In short, the naked virus gains an envelope.

This model, the result of data from multiple groups, leaves us with a new paradigm for picornavirus replication. These viruses, for so long thought to be cytoplasmic, may in fact be more infectious if engulfed in an organelle lumen. These so-called “naked viruses,” thought to be bare in the cytoplasm, may in fact swaddle themselves in multiple layers of membranes prior to cell lysis. And, just possibly, our work may reveal a replication strategy that can provide a mechanistic evolutionary link between the enveloped and nonenveloped viruses.
